# Sensitive Dentine in a Pulpless Tooth

**Published:** 1869-08

**Authors:** 


### SENSITIVE DENTINE IN A PULPLESS TOOTH.

YESTERDAY I was annoyed with "sensitive dentine," while having a tooth prepared for filling. The pulp has been dead since March. Passing broaches into the canals caused no pain; but every cut in the crown gave the same sensation as if the pulp had been alive, with more than the normal sensitiveness of the dentine. I account for the sensation in the same way that we explain a sense of tingling or pain in the fingers after the hand has been amputated. In "toothedge," or toothache in artificial teeth we have an analogous condition. In an adjacent pulpless tooth this sensation was not felt. W.

